# The expert’s cadence, the novice’s rush: A multi-level analysis of cognitive rhythm in translation production under pressure

**DOI:** 10.1371/journal.pone.0352322

**Published:** 2026-06-25

**Authors:** Yu Weng

**Affiliations:** Department of Language Science and Technology, The Hong Kong Polytechnic University, Hong KongChina; University of Missouri Columbia, UNITED STATES OF AMERICA

## Abstract

This study examines how time pressure and translator experience interact to shape the cognitive rhythm of translation production. Sixty-five Chinese-English translators’ (35 novices, 30 experienced) keystroke activities were tracked as they translated comparable texts under three time conditions: Short, Standard, and Free. Multi-level analyses captured micro-level behaviors (pausing, segmentation) and macro-level process organization (phase allocation, revision strategies). Results reveal experience-dependent adaptation. Both groups demonstrated increased fluency and production stability when time pressure was relieved, producing longer and more consistent text segments and engaging in more immediate self-correction. However, the two groups diverged fundamentally in their strategic responses. Experienced translators maintained stable production rhythms across all conditions and strategically allocated time to orientation and end revision, cleanly separating drafting from revision. Novices, in contrast, exhibited a reactive, blended strategy: drafting and revision were tightly coupled, and process efficiency depended heavily on external time constraints. Notably, novices’ behavioral pattern most closely resembled that of experienced translators under the moderate deadline, but they failed to capitalize on unlimited time for further strategic improvement. These findings suggest that translation expertise is characterized by metacognitive control over cognitive rhythm and resilience against external constraints. Novices, lacking such adaptive regulations, are prone to a cognitively costly, reactive process. The study’s implications extend beyond translation, highlighting the universal importance of strategic process management in professional practice and demonstrating how time constraints can serve as a pedagogical scaffold to help beginners develop adaptive expertise.

## 1. Introduction

Rhythm, a fundamental organizing principle structuring events in time, pervades both natural phenomena and human activities, shaping various cognitive functions such as language production and comprehension [1–3]. Inherently temporal, the rhythm of any cognitive activity is thus inseparable from the timeframe in which it unfolds. Consequently, time constraints may significantly influence the temporal dynamics of cognitive processing. As a complex cognitive activity, translation workflow exhibits rhythmic processing patterns manifested in multiple temporal scales, from millisecond-level keystroke patterns to broader phase transitions in the translation process. Time constraints serve as both an external frame and an internal modulator of the translation process. On a moment-to-moment basis, the varying time intervals between keystrokes reflect different cognitive and non-cognitive operations that may compress or expand under different timeframes, thus altering the cognitive rhythm [4]. In terms of broader patterns, translators’ allocation of time and cognitive resources across some sequential phases such as drafting and end-revision, adapts to the given temporal constraints [5]. Through these mechanisms, time constraints play a crucial role in shaping, and potentially dictating, the rhythm of cognitive processing throughout translation production.

Furthermore, given that professional experience modulates behavior through automated processes and efficient problem-solving strategies developed via extensive practice [6], experienced translators likely exhibit distinct rhythmic patterns compared to novices when working under time pressure as demonstrated in earlier research [7]. This experience-dependent temporal adaptation may reveal fundamental principles about skill acquisition in complex cognitive tasks and the development of temporal optimization strategies. While translation experience and professional status do not necessarily equate to true expertise — typically defined as consistently superior and adaptive performance developed through deliberate practice [8,9] — experience nonetheless provides a practical empirical lens through which expertise-related processing patterns can be identified.

This study systematically investigates the joint influence of time pressure and translator experience on the multi-level structure of cognitive rhythm in translation activity. Using the keystroke logging method, which provides high-resolution temporal data, both micro-level behavioral patterns (keystroke intervals) and macro-level process organization (time allocation across phases) are examined. By incorporating translation experience as a key variable, we aim to understand how differences associated with accumulated professional practice shape the impact of temporal constraints on cognitive processing rhythms—and what these differences reveal about the nature of translation expertise more broadly. This investigation will offer insights into the dynamic interplay of time, experience, and cognitive processing in translation, which represents a unique case of text production under dual-language processing demands.

## 2. Research background

### 2.1. Rhythm in text production

Text production, whether monolingual writing or the bilingual task of translation, is inherently a temporal endeavor. The unfolding of thoughts into written language is not a seamless, uniform stream but rather a dynamic, often cyclical, process characterized by its own distinct cognitive rhythm. Cognitive theories of writing posit that the creation of text is a multifaceted cognitive undertaking involving iterative cycles of core processes such as planning (generating ideas, setting goals, organizing), translating (transforming thoughts into linguistic form), reviewing (evaluating and revising the text), and monitoring the entire endeavor [10,11]. These cycles, influenced by individual intentionality and self-regulatory mechanisms [12,13], orchestrate the transformation of thoughts and ideas into written text. The inherent back-and-forth between generation, evaluation, and refinement, naturally gives rise to rhythmic patterns in cognitive processing [10,14].

Cyclic processing has also been widely conceptualized in models of translation. Nord’s [15: 38,39] looping model describes translation not as a linear progression from source to target, but as a circular, recursive process driven by an indefinite number of feedback loops, where translators consistently “look back” at previous steps to confirm or correct their analytical findings based on the target text’s overarching objective. Angelone [16] showed that translators engage in cyclic “problem-solving bundles” consisting of three metacognitive stages: problem recognition, solution proposal, and solution evaluation. Expert translators tend to cycle through these stages predictably, whereas novices often display disjointed behavior, shifting erratically between unresolved problems. Effective translation thus relies on bundled metacognitive regulation, whereby translators iteratively identify difficulties, test possible solutions, and evaluate their adequacy before proceeding to the next unit.

At a more granular level, cognitive rhythm also manifests in the temporal dynamics of physical text inscription, be it typing or handwriting. This sequential arrangement of letters and words, governed by linguistic conventions and modulated by individual skill and experience, inherently creates a nuanced temporal pattern characterizing the act of writing [17]. Research on skilled typewriting suggests that this pattern is supported by a hierarchical control system consisting of an “outer loop,” responsible for language generation, and an “inner loop,” responsible for motor execution [18]. Once a word-level representation is passed to the motor system, the inner loop automatically prepares the keystrokes for the entire word in parallel rather than processing each letter serially [19,20]. This division of labor enables rapid and fluent keystroke execution, providing the motor foundation for the rhythmic flow of text production. As motor execution proceeds automatically within the inner loop while higher-level planning occurs in the outer loop, writing naturally unfolds in “bursts” of rapid text production interspersed with pauses of varying durations as captured by keystroke-logging research (e.g., [21,22]). Further detailing these components, Chenoweth and Hayes [23] distinguished between P-bursts (segments terminated by pauses, often defined as >2 seconds) and R-bursts (those terminated by revisions or other discontinuities). The length of these bursts, their production speed, and their variability serve as indicators of a writer’s fluency, cognitive capacity, and efficiency in execution processes [21,24–28]. Research has consistently shown that expert writers tend to produce longer burst lengths [29], suggesting greater automaticity and processing efficiency.

The nature of these bursts, however, differs in important ways between monolingual writing and translation. In writing, bursts are typically interpreted as the execution of internally generated linguistic units reflecting the writer’s planning scope and production fluency [21,22]. In translation, by contrast, bursts are more strongly constrained by the segmentation of the source text, as translators must continuously coordinate source-text comprehension with target-text formulation. This is supported by research on cognitive segmentation which shows that translators divide the source text into manageable segments during processing (e.g., [30]) and by evidence of attention continuously shifted between source and target texts during production (e.g., [31]).

Far from being mere cessations of activity, the pauses that separate the bursts are considered critical windows into underlying cognitive processing. Early writing research suggested that short pauses might reflect lower-level lexical access or motor execution, while longer pauses are often associated with higher-order cognitive operations such as planning, sentence formulation, problem-solving, and revision [32,33]. However, increasing research suggests that pause duration may not map neatly onto specific cognitive operations. In translation, for instance, short- and mid-length pauses have also been shown to index heightened cognitive load traditionally associated with longer pauses, while higher-order processes are not confined to long pauses [34–37]. Therefore, rather than signaling discrete cognitive processes, pause distribution and duration are more appropriately interpreted as general indicators of fluctuations in cognitive effort and processing dynamics.

### 2.2. Rhythm in translation processes

As analyzed earlier, translation shares the fundamental cyclical and recursive nature with monolingual writing, but it differs from monolingual writing in involving a distinct process of interpreting the source text, transferring meaning, and restructuring it into a target text [38]. Whyatt et al. [39] conceptualize cognitive rhythm in translation as the temporal patterning of attention allocation and task coordination, as reflected in observable processing behaviors. This rhythmic dimension of translation can be examined at both macro and micro levels, each revealing different aspects of the cognitive dynamics involved.

At the micro level, Jakobsen [40: 112] describes how translators must integrate reading comprehension and text production into a “single, fairly regular rhythm,” with text construction progressing through the production of sizeable chunks at regular intervals. However, this basic rhythm, created by alternating reading/monitoring and typing activities, is frequently disrupted when translators encounter difficulties in comprehending source text meaning or finding appropriate target language expressions. These observations have led researchers to explore concepts such as segmentation [36,41], production units [42,43], and translation units [43,44]. These concepts typically refer to flows of successive text typing of varying size flanked by pauses, representing what Saldanha and O’Brien [45: 112] describe as “bursts of creativity in between pauses.”

Recent process-oriented research conceptualizes translation production as a sequence of task segments bounded by intentional pauses, within which translators engage in specific subtasks such as adding text, revising existing output, searching for information, or interacting with the interface [36]. Translating fluency therefore depends on how efficiently these subtasks are grouped and sequenced across segments rather than erratically interwoven. Empirical studies also show that the size and frequency of target-text production units vary according to cognitive constraints. For example, increased source-text difficulty tends to produce a greater number of smaller units, suggesting that translators segment the task more finely to manage cognitive load, with working memory further influencing unit size, although this influence may diminish as task difficulty increases [43]. Complementary evidence from task-comparison studies indicates that translation exhibits a particularly dense pattern of micro-level pauses, reflecting frequent lexical and syntactic processing demands triggered by the source text [46]. Overall, the rhythmic structure of translation production emerges from the dynamic coordination of subtasks such as comprehension, decision-making, and typing, resulting in pause–production cycles that reflect both cognitive load and strategic task management.

Despite widespread recognition of translation’s rhythmic processing features, the operationalization of these rhythms, particularly the definition of “pauses”, remains inconsistent in translation studies. Fixed pause thresholds of 3–5 seconds are commonly used to mark intensive cognitive processing, especially problem-solving activities (see a review in [47]). However, recent research advocates for more nuanced approaches based on individualized pause thresholds [34–37]. Applying such calibrated thresholds, studies suggest that higher-order processes such as planning and problem-solving occur not only during long pauses but also frequently during typing itself [36,37]; mid-length pauses can capture task-related cognitive activities beyond problem-solving, particularly monitoring processes associated with uncertainty and high cognitive load [34,35]; and individualized long-pause thresholds identify problem-solving instances more effectively than a fixed 3-second threshold [35].

The macro-level rhythmic patterns manifest in phase organization, namely how translators distribute time and cognitive resources across different phases of the translation process [39]. Jakobsen [48] operationalized these phases using keystroke logging, distinguishing among orientation, drafting, and end revision stages. The orientation phase, dominated by comprehension activities, serves as a crucial preliminary to subsequent text processing. The drafting phase, marked by the first keystroke of text production until the final punctuation mark, involves constructing meaning and producing target text, including “online” revisions. During the end revision phase, text production speed significantly decreases as the translator focuses on monitoring and refining the existing text. While the boundaries between phases may not always be clear-cut, this three-phase model provides a valuable framework for quantifying and comparing macro-level translation process dynamics.

Research shows that time constraints disproportionately affect the macro-rhythms of translation, making the orientation and end revision phases more vulnerable to compression than the drafting phase [4,5]. Experience also shapes these rhythmic patterns. Professionals, for instance, typically draft faster but allocate relatively more time to orientation and end revision compared to students [48]. However, other research indicates that students engage in more initial planning, while professionals allocate more time to final revisions and adopt a more locally-oriented drafting process, likely due to their ability to generate target text efficiently without extensive reference beyond the immediate co-text [49].

At the micro-rhythm level, studies also reveal patterns associated with experience. For example, early research found that professionals tend to work with fewer but longer text segments, establishing equivalence at the clause level, whereas less experienced translators operate more at the phrase level [30]. Yet, this finding has been complicated by a more recent study, which found that experience level did not significantly affect the average size of translation units [50]. The authors attribute this discrepancy to the different translation directions used in their respective studies.

### 2.3. Cognitive strategies and their rhythmic signatures

The cognitive rhythm of text production offers a window into the underlying strategies employed by writers and translators. Scardamalia and Bereiter’s [51] influential models of composing, Knowledge Telling and Knowledge Transforming, provide a valuable framework for understanding these strategic differences and their manifestation in rhythmic patterns.

In general text production, the Knowledge Telling strategy characterizes writing as a more straightforward, less problematic activity, typically exhibiting a steady, predictable rhythm, with largely linear text production and minimal disruptions for revision or problem-solving. Writers employing this approach make “maximum use of existing cognitive structures and minimize the extent of novel problems that must be solved” [51: 5]. This strategy relies heavily on retrieval from memory and familiar discourse structures, marked by observable rhythmic signatures such as infrequent online revisions, linear text production, and a stable ratio between pausing and production [32]. In contrast, the Knowledge Transforming strategy represents a more effortful, problem-solving approach to writing. With this strategy, writers actively reprocess and transform their existing knowledge to address novel challenges they encounter during composition [51: 11]. This recursive process involves continual negotiation between content and rhetorical considerations, leading to deeper engagement with both the material and the writing task itself. Consequently, it tends to produce a more variable rhythmic pattern characterized by nonlinear progression, frequent revisions, and a less stable relationship between planning and production phases.

In translation, however, the task differs in that translators work primarily with knowledge already expressed in the source text rather than generating new content. In this context, the distinction between Knowledge Telling and Knowledge Transforming can be interpreted more broadly as reflecting two modes of processing: routine reformulation based on readily available linguistic equivalents, and effortful problem-solving when such reformulation cannot adequately address translation challenges. Thus, in translation production, Knowledge Telling can be understood as relatively automatic retrieval and reformulation of source-text meaning, whereas Knowledge Transforming corresponds to more deliberate problem-solving processes, such as resolving ambiguities, restructuring syntax, or adjusting rhetorical effects to achieve functional equivalence. These patterns echo, to some extent, Tirkkonen-Condit’s [52] monitor model of translation, in which a default, automatic “horizontal” process is only intermittently interrupted by a conscious, controlled “vertical” monitoring process when problems arise. However, as evidenced by emerging empirical findings, translation production is far more complex than a binary automaton switching between two processing modes; rather, it represents an uneven continuum of intermittent, alternating subtasks [34]. Thus, instead of adopting a binary perspective, we treat Knowledge Telling/Transforming as describing strategic orientations, which can be complemented by more nuanced process signatures of how these orientations manifest behaviorally.

These models have provided insight into experience-related behavioral differences in the translation process. Jensen [4] revealed that professional translators demonstrate greater flexibility in their cognitive approaches. When confronted with translation problems, professionals more frequently employ Knowledge Transforming behaviors than their non-professional counterparts, engaging in deeper processing and more sophisticated problem-solving. Interestingly, professionals also exhibit greater proficiency in utilizing the Knowledge Telling strategy when appropriate, suggesting that expertise in translation involves not just more complex processing capabilities but also the strategic wisdom to determine when such complexity is warranted. This adaptive application of different cognitive strategies, each with its distinctive rhythmic signature, represents a hallmark of translation expertise.

### 2.4. The present study

The preceding review shows that cognitive rhythm is a fundamental aspect of text production, including translation, manifesting at both macro (e.g., phase distribution, revision pattern) and micro levels (e.g., pause-burst dynamics reflecting cognitive operations). This rhythm could be shaped by time constraint and translator experience. However, the literature has key limitations: many early studies are constrained by small sample sizes, most micro-rhythm analyses rely on fixed pause thresholds that limit sensitivity to individual differences, and there is little integration of micro- and macro-level metrics to provide a comprehensive understanding of the rhythmic nature of translation production. Additionally, while the individual influences of experience and time constraints on certain rhythmic aspects have been explored, the interaction between these key factors on rhythmic patterns in translation has rarely been systematically examined.

Employing the keystroke logging method, this study aims to extend our understanding of how time constraints and translator experience jointly shape cognitive rhythm in translation at two levels. At the micro-level, it analyses the pausing behavior and text segmentation, following the framework of individualized pause threshold in Muñoz Martín & Martín de León [34]. At the macro-level, it examines the allocation of time and effort across translation phases and revision patterns to understand strategic adjustments influenced by time and experience. Specifically, the study addresses the following research questions (RQs):

RQ1. How do time constraints and translation experience jointly affect micro-level (pausing and segmentation) and macro-level cognitive rhythm (translation phases and revision patterns) during translation production?

RQ2. How does the rhythmic patterns reflect shifts in cognitive strategies (i.e., Knowledge Telling and Knowledge Transforming) under time pressure for novice and experienced translators?

## 3. Methods

### 3.1. Participants

Sixty-five participants were voluntarily recruited (May 05, 2019–March 01, 2020) after the study received ethical approval from the ethics committee of Durham University. All participants were touch-typists and native Chinese speakers with English as their second language. These participants were divided into two groups. The novice group included 35 postgraduate Translation Studies students from a UK university with no professional experience (5 males, 30 females; age range: 21–34 years, M = 24.14, SD = 2.68). The experienced group comprised 30 freelance or in-house translators with 3–23 years of English-Chinese translation experience (M = 7.23, SD = 5.47; 9 males, 21 females; age range: 24–50 years, M = 30.5, SD = 8.02). All participants received monetary compensation for their time and effort.

### 3.2. Source texts

Three English texts on the China-US trade war were excerpted from *The Economist*, each containing 11 sentences and averaging 201 words (SD = 5.51). The texts were confirmed to be comparable through objective and subjective evaluations. Objectively, they were balanced on six major readability indices and a word frequency metric: each word in each text was examined in the British National Corpus, and words which appeared fewer than 1000 times (i.e., the frequency value) in the corpus were marked as “Frequency 1000”; the word frequency index in this study thus refers to: [(Frequency 1000 ÷ the number of total unrepeated words of the text)×100%]. Subjectively, a pre-test involving 21 independent translators (eight experts, 13 students) rated the texts on comprehensibility, translatability, and translation difficulty. Both evaluation methods concluded that the source texts were comparable and of medium difficulty (see S1 File).

### 3.3. Time constraints

This study manipulated three time conditions: Short (16 min 15 s), Standard (20 min 25 s), and Free (unlimited). Timeframes for the Short and Standard conditions were derived from the first quartile and median, respectively, of untimed translation durations recorded for the source texts by 13 pre-test participants (student translators) in a laboratory setting.

### 3.4. Procedures

Prior to the experiment, all participants signed a written consent form and completed a background information questionnaire. To familiarize them with the experiment, a warm-up session was held, during which participants practiced using the laboratory interfaces and devices. At this stage, a Chinese touch-typing test was also administered; results showed no statistically significant difference in typing speed between the two groups (average keydown-to-keydown interval: novices, *M* = 317.63 ms, *SD* = 75.27; experienced translators, *M* = 314.28 ms, *SD* = 65.95; *t*(63) =.19, *p* = .851).

After the warm-up session, each participant completed three English-Chinese translation tasks using Translog II [53], a keystroke logging software designed for translation tasks, which provides separate source and target text panels and records all keystrokes during the translation process. In the interface, the source text appeared at the top of the screen, with the target text editing box displayed below. A countdown timer (for Standard and Short sessions) or a stopwatch (for the Free session) was shown at the bottom to indicate the remaining or elapsed time. Participants were not permitted to consult any online or offline resources during the translation tasks. However, a printed glossary of the seven most unfamiliar words in each source text, as identified during the pre-test, was provided. Participants were allowed to consult resources about these words before each task. The texts and time conditions were counterbalanced to minimize order effects.

### 3.5. Statistical analysis methods

Statistical analysis was performed using Linear Mixed-effects Regression (LMER) modelling with the *lmer4* package [54] in R (version 3.6.3) [55]. Percentage-based measures were analyzed using beta regression (generalized linear mixed model, GLMM) with the *glmmTMB* package [56], which is well-suited for modelling data bounded between 0 and 1. Separate LMER or GLMM models were constructed for each target measure, including Condition (Short, Standard, and Free) and Group (Novice and Experienced) as fixed effects. Interaction terms were included only if they were (marginally) significant; otherwise, only the main effects were retained in each model. Individual variability was accounted for by including participant as a random effect. The residuals of each model, or their log-transformed versions, conformed to a normal distribution. P-values for the fixed effects were calculated using Satterthwaite’s approximation provided by the *lmerTest* package [57].

### 3.6. Metrics

The measures analyzed in the present study were based on the minimum units of target text production, i.e., the typing units (TUs) of Chinese, to reflect cognitive rhythm in translation at different levels. A typing unit is defined as a string of keystrokes starting with a pinyin letter keystroke, ending with the (first) confirmation keystroke for typing out the Chinese character(s). The interval within a TU (IwTU) and between a TU (IbTU) were used to define the individualized pause thresholds. Although Translog II served as the interface for presenting the source and target texts, keystroke metrics were calculated from data provided by the eye-tracking software Tobii Pro Studio, which offers direct timestamped keystroke data that Translog II does not; eye movement data collected simultaneously via Tobii TX300 are reported separately [6]. Following Muñoz Martín and Cardona Guerra’s [35] method, the lower threshold for identifying pauses is [2 × the median IwTU of a translation task]; the upper threshold is [3 × the median IbTU of a translation task]. Pauses between two successive keystrokes that last between 200 milliseconds and the lower threshold are classified as Short Pauses (SP), those between the lower and upper thresholds as Mid Pauses (MP), and those exceeding the upper threshold as Long Pauses (LP). In line with Muñoz Martín and Martín de Leon [34], LPs are used to define Segments, which consist of all activities (keystrokes, SPs, and MPs) occurring between two LPs.

Translation phases were determined based on Jakobsen’s [48] framework as mentioned in Section 2.2. Deletions during drafting were taken to represent online correction and formulation processes. Deletions during the end revision phase were considered as indicators of offline text polishing and editing. The details of all the measures analyzed are presented in Table 1.

**Table 1:** Summary of the measures analyzed in this study.

Construct	Metric	Description	Operational definition/ Calculation
**Pauses**	Pause Rate	The frequency of pausing during the task, calculated per minute.	(Total count of a given pause type¹)/ (Total task duration in minutes)
	Pause Density	The number of pauses relative to the amount of text produced.	(Total count of a given pause type¹)/ (Total TUs produced)
	Pause Time Proportion	The proportion of total task time spent in a non-productive, paused state.	(Total duration of a given pause type¹)/ (Total task duration)
**Production Units (Segments)**	Segment Length (Duration)	The average time elapsed between two consecutive long pauses.	Mean duration (in ms) of all segments.
	Segment Length (TU count)	The average number of TUs produced between two consecutive long pauses.	Mean number of Typing Units (TUs) per segment.
	Segment Stability	The consistency of the production rhythm. Lower values indicate a more stable pace.	Standard Deviation (SD) of segment durations and/or segment lengths.
	Segment Production over Time	The rate of segment production across the task, used to model changes in fluency.	Count of segments produced within sequential time bins (5% intervals of total task time) for Growth Curve Analysis.
**Task Phases**	Phase Duration Proportion	The proportion of total task time allocated to each of the three main translation phases.	(Duration of a specific phase²)/ (Total task duration)
**Revision Behavior**	Online Revision Rate	The extent of corrective behavior during the drafting phase, capturing immediate self-monitoring.	Total count of deletion keystrokes^3^ logged during the Drafting phase.
	End Revision Rate	The extent of deliberate editing and polishing after the initial draft is complete.	Total count of deletion keystrokes^3^ logged during the End Revision phase.

Notes: ¹Calculated separately for Short Pauses (SP), Mid Pauses (MP), and Long Pauses (LP) based on predefined duration thresholds. ²Phases include Orientation, Drafting, and End Revision. ^3^The deletion keystrokes for online and end revision refer to those fall outside the TUs, such as deleting Chinese characters; deletions of Pinyin letters within TUs are not counted, as they may reflect technical typing errors rather than cognitive revision processes.

## 4. Results

### 4.1. Pauses

Analysis of pause measures revealed a broad impact of time pressure (Condition), whereas the effect of experience (Group) was concentrated in LPs through interactions with time pressure (Table 2).

**Table 2:** Summary of the models for pause measures.

	Pause Rate (count/min) (LMER)			Pause Density (count/TU) (LMER)			Pause Time Proportion (GLMM)		
Predictor	SP	MP	LP	SP	MP	LP	SP	MP	LP
(Intercept)	20.34(1.48)***	20.16(.82)***	3.67(.23)***	1.75(.16)***	1.62(.07)***	.28(.02)***	−2.37(.12)***	−.93(.07)***	−.32(.07)***
**Condition**									
Standard	−1.49(.47)**	−2.6(.42)***	.01(.18)	.03(.03)	−.02(.04)	.04(.01)***	−.07(.03)*	−.18(.04)***	.26(.04)***
Free	−.97(.47)*	−2.75(.42)***	−.08(.18)	.17(.03)***	.05(.04)	.03(.01)*	−.04(.03)	−.13(.03)***	.21(.04)***
**Group**									
Experienced	−0.67(2.14)	−.43(1.15)	.70(.35)*	−.20(.23)	−.15(.11)	.03(.02)	−.04(.17)	−.11(.1)	.04(.10)
**Condition × Group**									
Standard × Experienced	---	---	−.54(.27)*	---	---	−.03(.02).	---	---	---
Free × Experienced	---	---	−.65(.27)*	---	---	0(.02)	---	---	---
**Random effects (SD)**									
Subject	8.48	4.39	1.17	.94	.41	.08	.67	.39	.36
Residual	2.69	2.41	.76	.20	.21	.05	---	---	---

Note: Values are *β* (SE). The baseline for Condition is “Short” and for Group is “Novice”. Interaction terms were included only when marginal (.05 ≤ *p* < .10) or significant (*p* < .05) effects emerge. Significance codes:. *p* < .10, * *p* < .05, ** *p* < .01, *** *p* < .001.

For both groups, relaxing time constraints reduced the rate and time proportion of short and medium pauses, indicating greater fluency. This additional time was reinvested into more deliberate processing, as participants significantly increased the density of short and long pauses per unit of text and dedicated a greater proportion of time to long pauses. Notably, while the rate of SPs decreased, their density relative to text output (count per TU) significantly increased in the Free condition, suggesting more fine-grained monitoring when time was not a factor.

**Fig 1:**
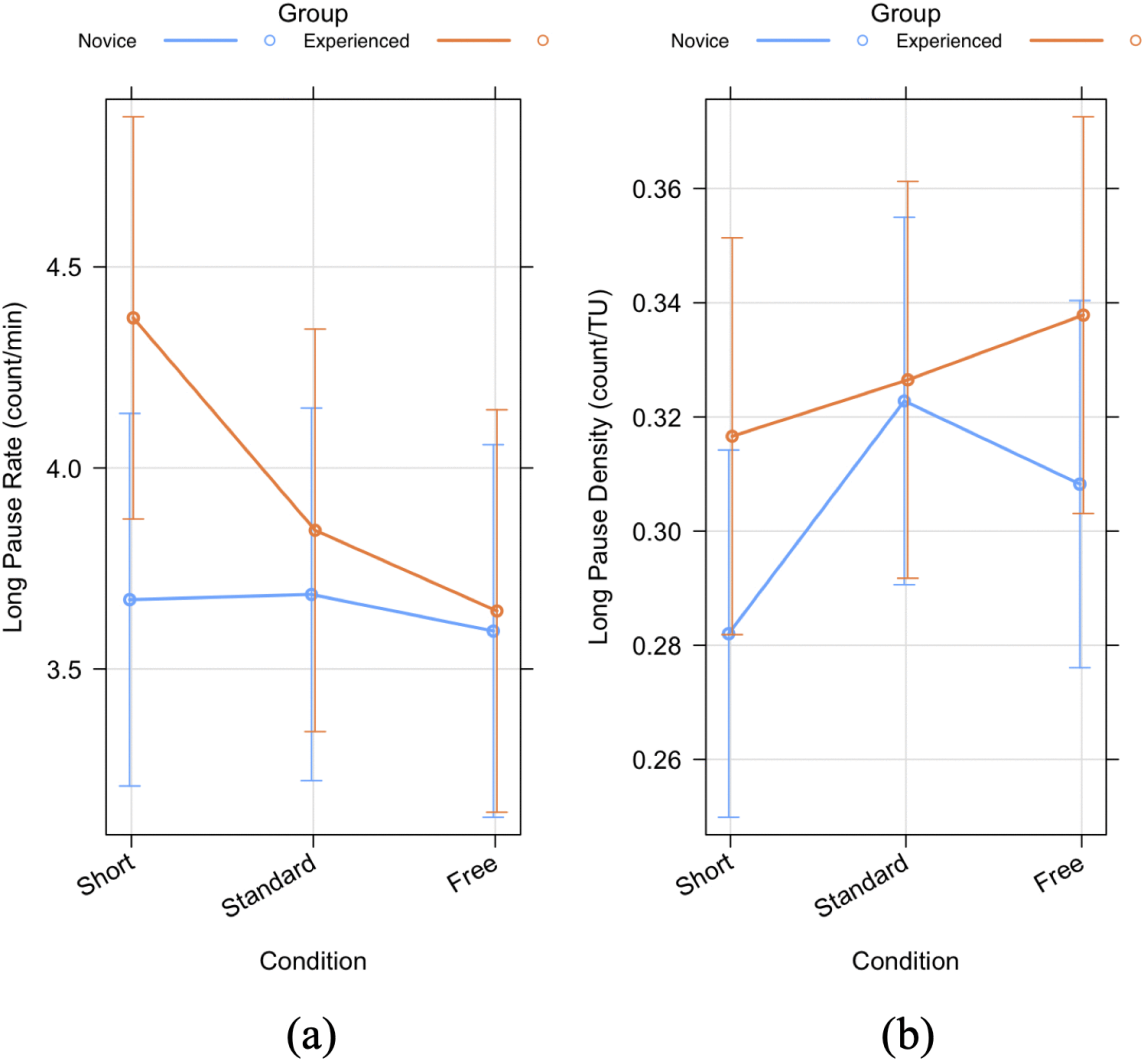
Interactional effects of Condition and Group on LP rate (a) and LP density (b).

On the other hand, the strategies for LPs diverged significantly across groups (Fig 1). Experienced translators exhibited a high rate of LPs under severe time pressure but markedly decreased this rate as time became available. Novices, however, maintained a consistently low LP rate across all conditions. A marginal interaction between Group and Condition was observed for LP density. As shown in Fig 1, experienced translators maintained a relatively high and stable density of LPs across all conditions. In contrast, novice translators’ LP density increased sharply from the Short to the Standard condition, nearly closing the gap with the experienced group, before declining in the Free condition. This tendency demonstrates a temporary strategic alignment under moderate time pressure.

### 4.2. Segments

**Fig 2:**
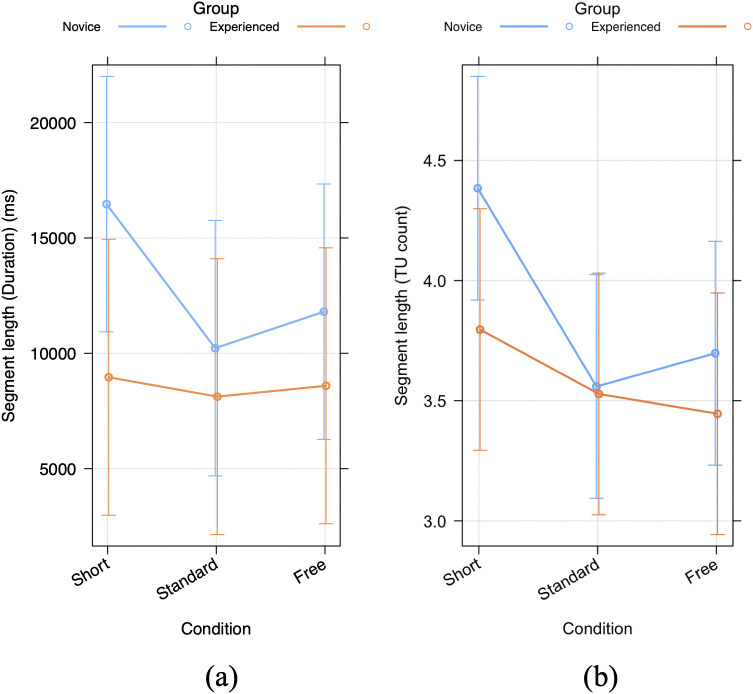
Interactional effects of Condition and Group on segment length (duration) (a) and segment length (TU count) (b).

Analysis of segment-level production revealed a significant interaction between Condition and Group on both segment duration and length in TUs (Table 3). This interaction is visualized in Fig 2, which shows that novice translators significantly shortened their segments particularly in the Standard condition, whereas experienced translators maintained relatively stable segment lengths across all three conditions. This suggests that novices altered their basic production unit size in response to time constraints.

**Table 3:** Summary of the segment measure models.

	Segment length (LMER)		Segment variation (LMER)	
Predictor	Duration (log)	TU count (log)	Duration SD (log)	TU count SD (log)
(Intercept)	9.21(.10)***	1.39(.05)***	9.13(.10)***	1.34(.06)***
**Condition**				
Standard	−.19(.06)***	−.16(.04)***	−.12(.05)**	−.10(.03)**
Free	−.06(.06)	−.11(.04)**	−.07(.05)	−.13(.03)***
**Group**				
Experienced	−.26(.14).	−.11(.07)	−.014(.16)	−.04(.08)
**Condition × Group**				
Standard × Experienced	.17(.08)*	.11(.05)*	---	---
Free × Experienced	.07(.08)	.03(.05)	---	---
**Random effects (SD)**				
Subject	.53	.25	.58	.29
Residual	.23	.15	.26	.19

Note: Values are *β* (SE). The baseline for Condition is “Short” and for Group is “Novice”. Interaction terms were included only when marginal (.05 ≤ *p* < .10) or significant (*p* < .05) effects emerge. Significance codes:. *p* < .10, * *p* < .05, ** *p* < .01, *** *p* < .001.

In contrast, segment variation was affected only by a main effect of Condition. For both segment duration and segment length in TU count, relaxing time constraints led to less variations for all participants. The lack of a significant Group effect or interaction indicates that both novices and experienced translators achieved greater production stability when time pressure was alleviated.

### 4.3. Production rhythm over time

**Fig 3:**
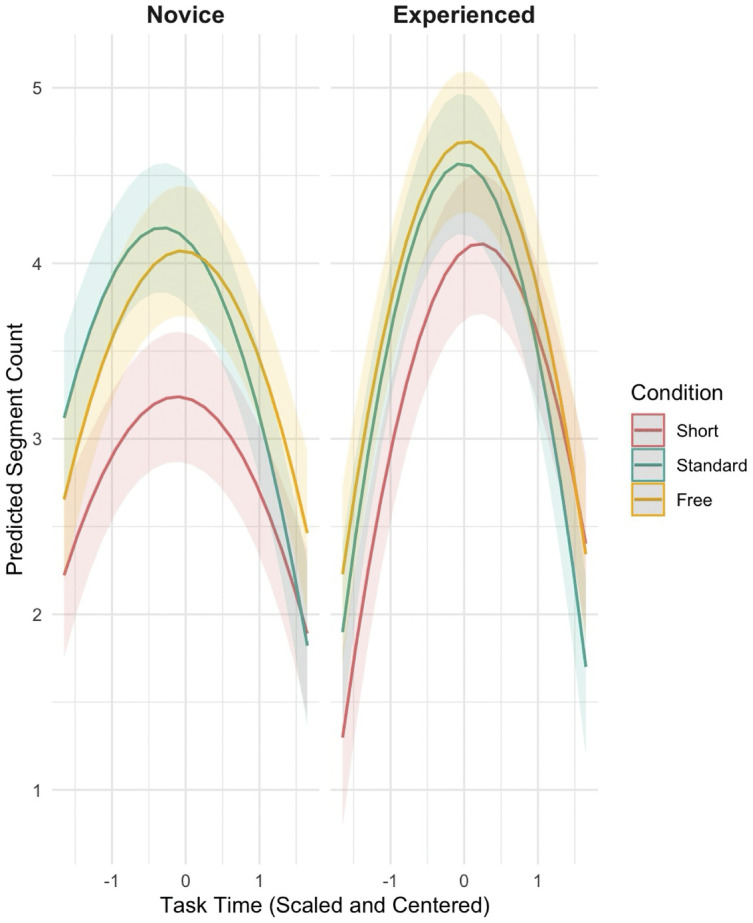
Effects of Time, Condition and Group on production rhythm (segment count over time).

To investigate how production rhythm, i.e., segment count, evolved during the task, a Growth Curve Analysis (GCA) was performed. The model revealed a significant three-way interaction between Time (linear and quadratic), Condition, and Group (Table 4; Fig 3), indicating that the production trajectory of experienced translators and novices differed depending on the level of time pressure.

**Table 4:** Summary of the growth curve analysis on segment count.

Predictor	Segment count (LMER)
(Intercept)	3.23(.19)***
**TimeBin_cs**	−.10(.06)
**I(TimeBin_cs^2)**	−.43(.07)***
**Condition**	
Standard	.91(.14***
Free	.84(.14)***
**Group**	
Experienced	.84(.28)**
**TimeBin_cs × Condition**	
TimeBin_cs **×** Standard	−.29(.09)**
TimeBin_cs **×** Free	.04(.09)
**I(TimeBin_cs^2) × Condition**	
I(TimeBin_cs^2) **×** Standard	−.18(.10).
I(TimeBin_cs^2) **×** Free	−.12(.10)
**TimeBin_cs × Group**	
TimeBin_cs **×** Experienced	.44(.09)***
**I(TimeBin_cs^2) × Group**	
I(TimeBin_cs^2) **×** Experienced	−.39(.11)***
**Condition × Group**	
Standard **×** Experienced	−.42(.20)*
Free **×** Experienced	−.22(.20)
**TimeBin_cs** **× Condition × Group**	
TimeBin_cs **×** Standard **×** Experienced	−.10(.13)
TimeBin_cs **×** Free **×** Experienced	−.34(.13)*
**I(TimeBin_cs^2) × Condition × Group**	
I(TimeBin_cs^2) **×** Standard **×** Experienced	−.02(.15)
I(TimeBin_cs^2) **×** Free **×** Experienced	.06(.15)
**Random effect (SD)**	
Subject	.96
Residual	1.7

Note: Values are *β* (SE). TimeBin_cs = centered time bins (the linear term for time); I(TimeBin_cs^2) = the quadratic term for time. The baseline for Condition is “Short” and for Group is “Novice”. Significance codes:. *p* < .10, * *p* < .05, ** *p* < .01, *** p < .001.

Fig 3 reveals crucial differences in pacing and production rhythm between experienced and novice translators, despite the observation that experienced translators consistently had a higher peak than novices across time conditions. The experienced group exhibits a more concentrated and intense pattern of production; their output ramps up quickly to a sharp, high peak near the task’s midpoint before declining steeply, suggesting a focused burst of high-efficiency work. In contrast, novices demonstrate a more tentative and distributed pacing, characterized by a broader, flatter production curve with a more gradual increase and a less dramatic drop-off.

Furthermore, the two groups responded differently to the relaxation of time constraints. The production peak for experienced translators progressively increased with more available time, reaching its highest point in the Free condition. Their pacing proved robust, with the Standard and Free condition curves being nearly indistinguishable. Novices, however, achieved their highest production peak in the Standard condition, and their output slightly decreased in the Free condition, with each time constraint eliciting a more distinct production rhythm. This divergence suggests that while experienced translators strategically leveraged unlimited time for greater productivity, novices may not adapt as effectively to the absence of time pressure.

### 4.4. Translation phases

**Fig 4:**
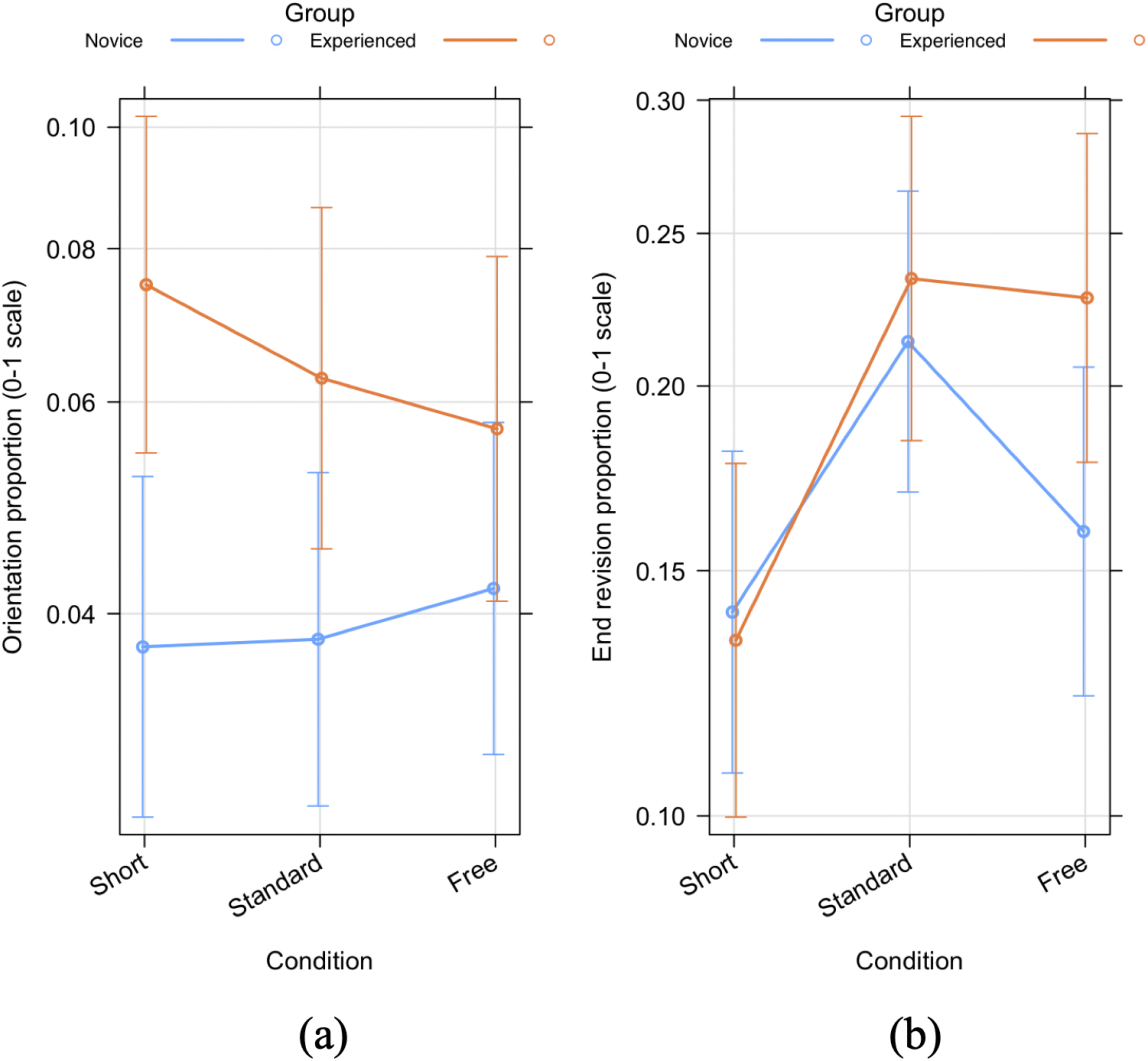
Interactional effects of Condition and Group on Orientation (a) and End revision time proportion (b).

The results on time allocation strategies for different translation phases in Table 5 and Fig 4 show that there was an interaction effect between Condition and Group on the proportion of the orientation and the end revision time. The drafting phase was mainly affected by Condition, and marginally affected by Group.

**Table 5:** Summary of translation phase models.

Predictor	Orientation (%) (GLMM)	Drafting (%) (GLMM)	End Revision (%) (GLMM)
(Intercept)	−3.25(.17)***	1.41(.11)***	−1.81(.15)***
**Condition**			
Standard	.02(.14)	−.39(.09)***	.51(.16)**
Free	.12(.14)	−.22(.09)*	.15(.17)
**Group**			
Experienced	.73(.24)**	−.27(.14).	−.05(.23)
**Condition × Group**			
Standard × Experienced	−.2(.18)	---	.17(.23)
Free × Experienced	−.41(.18)*	---	.49(.24)*
**Random effects (SD)**			
Subject	.81	.48	.58

Note: Values are *β* (SE). The baseline for Condition is “Short” and for Group is “Novice”. Interaction terms were included only when marginal (.05 ≤ *p* < .10) or significant (*p* < .05) effects emerge. Significance codes:. *p* < .10, * *p* < .05, ** *p* < .01, *** *p* < .001.

For the Orientation phase, a significant main effect of Group showed that experienced translators dedicated a significantly higher proportion of time to orientation than novices did, especially in the high-pressure Short condition. However, a significant interaction (Fig 4a) revealed that this gap between experienced translators and novices narrowed when time constraints were removed (Free condition), with novices increased and experienced translators reduced the proportional effort in the upfront planning phase.

In the Drafting phase, the results were primarily driven by the main effect of Condition, despite the fact that novices marginally invested a higher proportion of time than experienced translators in this phase. Compared to the Short condition, participants significantly lowered the proportion of drafting time in both the Standard and Free conditions. This indicates that when time pressure was reduced, participants shifted their effort away from pure text production, though this does not necessarily mean that they spent less absolute time on drafting. No significant interaction was found, suggesting both groups adjusted their drafting time proportion similarly across conditions.

Finally, for the End Revision phase, a significant interaction between Condition and Group emerged. Under the high-pressure Short condition, both groups dedicated a similarly low proportion of their time to revision. In the Standard condition, both groups increased their revision proportion to its highest point, with experienced translators revising slightly more than novices. The sharpest divergence emerged in the Free condition: the Novice group’s revision proportion dropped significantly back down to a level similar to the high-pressure Short condition; in contrast, the Experienced group maintained a high revision proportion. This indicates that experienced translators strategically use the availability of extra time to maintain a post-drafting polishing process, a behavior not observed in the novice group.

### 4.5. Types of revision behavior

Online and end revision behaviors were examined with the indicator of deletion count during the drafting and the end revision phase respectively. Table 6 shows that revision behavior during the drafting phase was primarily influenced by Condition, with no significant main effect of Group or interaction effects. Compared to the Short condition, the number of deletions increased significantly when participants were given unlimited time in the Free condition. The Standard condition did not differ significantly from the Short condition. This suggests that when time pressure was removed, all participants, regardless of group, engaged in more immediate, online correction and reformulation of their text as they typed.

**Table 6:** Summary of revision models.

Predictor	Online revision (deletion no.) (LMER)		End revision (deletion no.) (LMER)
(Intercept)	66.59(7.65)***		12.4(3.3)***
**Condition**			
Standard	4.45(3.84)		5.8(3.42).
Free	12.85(3.84)**		6.37(3.42).
**Group**			
Experienced	−3.54(10.78)		−1.53(4.86)
**Condition × Group**			
Standard × Experienced	---		3.3(5.04)
Free × Experienced	---		13.56(5.04)**
**Random effect (SD)**			
Subject	41.42		13.27
Residual	21.89		14.32

Note: Values are β (SE). The baseline for Condition is “Short” and for Group is “Novice”. Interaction terms were included only when marginal (.05 ≤ *p* < .10) or significant (*p* < .05) effects emerge. Significance codes:. *p* < .10, * *p* < .05, ** *p* < .01, *** *p* < .001.

**Fig 5:**
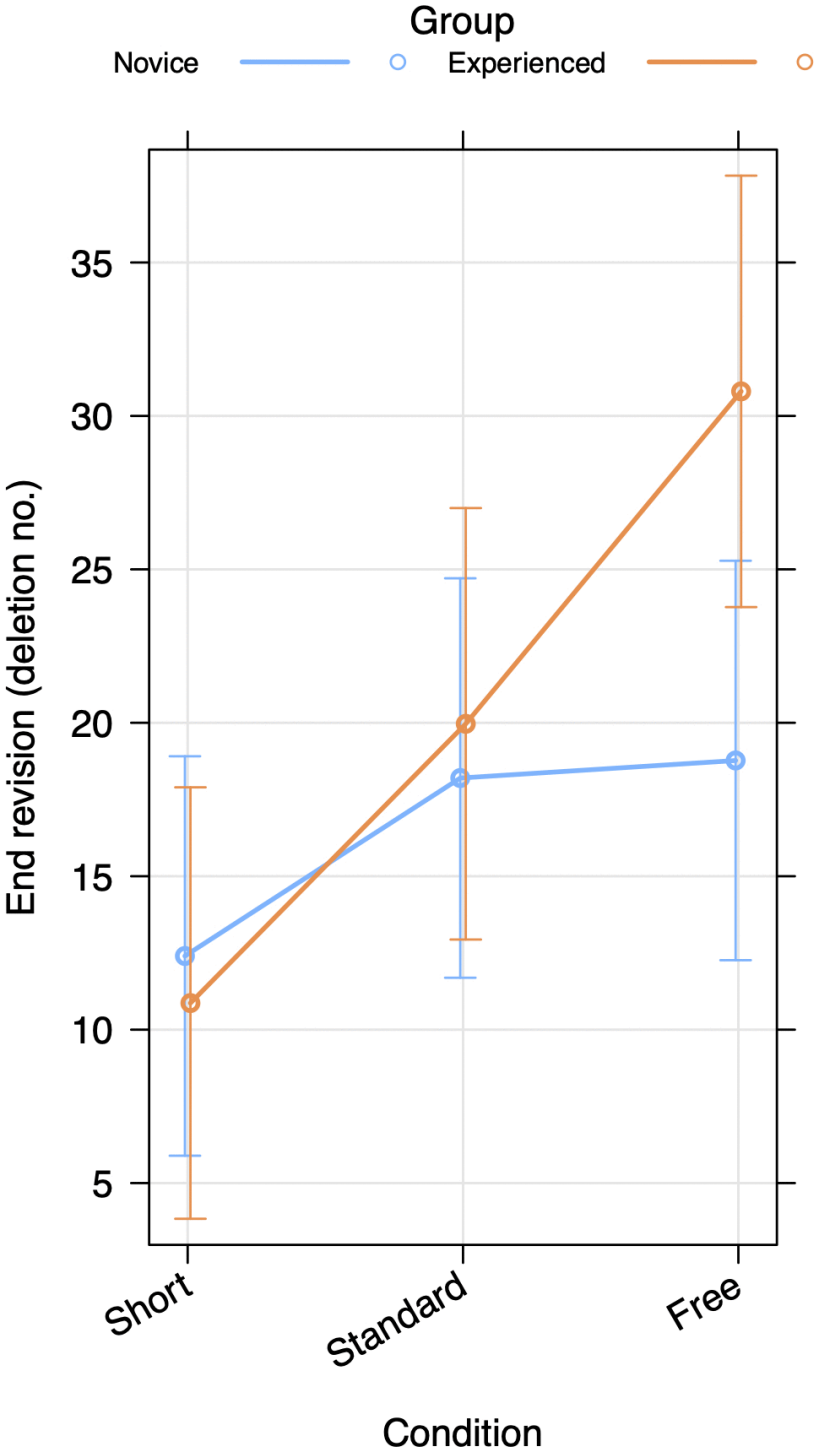
Interactional effects of Condition and Group on end revision deletions.

In contrast, the end revision phase was characterized by a significant interaction between Condition and Group (Fig 5). For novices, moving from the Short to the Standard or Free condition resulted in only a slight increase in deletions. Their end revision activity was not strongly affected by time pressure. The key finding is a significant interaction for experienced translators in the Free condition. This indicates that while experienced translators behaved similarly to novices under time pressure, they uniquely capitalized on the Free condition by dramatically increasing their number of deletions. They engaged in significantly more substantial post-drafting editing and revision when time was not a constraint.

### 4.6. Correlation analysis of revision and pausing behaviors

To further illuminate the complex relationships and potential mechanisms underlying different types of activities during translation production, pairwise Pearson’s correlation coefficients were calculated on different pausing behaviors and revision efforts for the two groups. The statistically significant results of this analysis are summarized in Table 7, revealing distinct strategic patterns for the novice and experienced groups. A primary distinction was found in the relationship between drafting deletions and end-revision deletions. A significant moderate positive correlation emerged for the novice group (*r* = 0.45, *p* < .001), while this relationship was weak and non-significant for the experienced group (*r* = 0.13, *p* > .05).

**Table 7:** Comparative correlation analysis between novice and experienced group.

Correlation Pair	Novice Group (*r*)	Experienced Group (*r*)
Online revision (deletion no.) vs. End revision (deletion no.)	0.45***	0.13
Online revision (deletion no.) vs. MP Duration (Drafting phase)	0	0.54***
End revision (deletion no.) vs. MP Duration (Drafting phase)	−0.23*	0.02
End revision duration vs. LP Duration (Drafting phase)	−0.07	−0.25*

Note: Significance codes: *** *p* < 0.001, * *p* < 0.05

Furthermore, the connection between online deletions and pausing differed significantly. The experienced group exhibited a moderate-to-strong positive correlation between draft deletions and the duration of mid-pauses (*r* = 0.54, *p* < .001). This correlation was absent in the novice group (*r* = 0).

Finally, pausing behavior during drafting showed weak negative correlations with subsequent revision effort in different ways for each group. For novices, the duration of MPs was weakly negatively correlated with the end-revision deletion count (*r* = −0.23, *p* < .05). For experienced translators, the duration of LPs was weakly negatively correlated with the final revision duration (*r* = −0.25, *p* < .05).

## 5. Discussion

This study demonstrates that professional experience is the critical factor that transforms adaptive pacing into an act of strategic process control. While both novice and experienced translators responded to the removal of deadlines with a slower, more meticulous rhythm, this surface-level similarity masks a fundamental divergence in process management. The following sections will synthesize the findings by addressing the study’s research questions. Section 5.1 and 5.2 will detail the joint influence of time pressure and translator experience on micro- and macro-level rhythms (RQ1), while Section 5.3 will explain how these rhythmic patterns serve as behavioral signatures of underlying cognitive strategies (RQ2).

### 5.1. The joint influence on micro-level rhythms

At the most granular level, the findings demonstrate that professional experience fundamentally shapes how translators adapt their micro-level rhythms to time pressure. The joint influence of these two factors is evident in both pausing and segmentation strategies, which are not mere byproducts of pacing but function as active indicators of cognitive control.

Pausing behavior clearly illustrates this interaction. While both groups reduced their rate of short and medium pauses when time was relaxed—a universal response to decreased pressure—their use of LPs diverged significantly. Experienced translators’ high LP rate under severe pressure (Fig 1) reflects a controlled, strategic deployment of effort to engage in higher-order planning or problem-solving even when time is scarce (cf. [34,47]). Novices, unable to employ this strategy, maintained a consistently low LP rate. Furthermore, novices’ LP density peaked only under a moderate deadline (Fig 1), suggesting an external, moderate pressure was needed to push them into a more strategic mode. This reliance on external “scaffolding” [58–59] highlights their lack of internalized control, which became evident again when their LP density declined in the Free condition.

Segmentation analysis provides an even clearer window into the joint effects of translator experience and time pressure on cognitive stability. The interaction effect (Fig 2) was stark: experienced translators exhibited a remarkably steady rhythm, with segment length remaining relatively consistent across all time conditions. This finding extends Dragsted’s [30] core observation of a consistent, higher-level processing strategy in professionals by revealing its resilience against external stressors, which serves as a robust marker of expertise and automaticity [6,60]. Additionally, this stability likely reflects metacognitive regulation, whereby experienced translators are well aware of their optimal processing tempo and able to maintain this chunking strategy consistently (cf. [16]). In contrast, novices displayed a reactive rhythm. Their segment length fluctuated significantly with time constraints, temporarily approximating an expert-like cadence under the moderate deadline, but this discipline vanished once the pressure was removed. This finding suggests that their production process is inherently vulnerable, relying on resource‐intensive operations dictated by external constraints rather than on the stable, internalized control that marks expertise.

### 5.2. The joint influence on macro-level rhythms

At the macro level, a clear split between the experienced translators’ strategic resource allocation and the novices’ reactive processing emerged. The interaction between translator experience and time pressure governed the entire organization of the translation process.

The divergence between experienced translators and novices is apparent in the allocation of time across task phases (Fig 4). Experienced translators demonstrated high-level cognitive control, strategically reallocating their temporal focus as conditions changed. Aligning with existing research [48], they compressed the proportion of time drafting to fund orientation and, critically, end revision when time allowed. For orientation, this pattern may partly reflect the use of a relatively short text (≈200 words), which allowed experienced translators to spend about 6–8% (Fig 4a) of the total task time across the three time conditions reviewing the text before drafting. In contrast, novices allocated only around 4% of the time, suggesting a less thorough initial reading and overview of the text. The significant interaction effect in the End Revision phase is particularly telling: experienced translators maintained a high revision time proportion in the Free condition, treating it as a distinct and vital stage. Novices, conversely, failed to reinvest their time in this way; their end revision effort dropped in the Free condition, suggesting weaker metacognitive planning and a failure to capitalize on available resources.

This strategic divide is reinforced by revision edits (Fig 5). While both groups engaged in more online corrections in the Free condition, only experienced translators significantly increased their end-revision deletions. This suggests that they use extra time for a deliberate, global post-drafting phase that is cognitively demanding and carefully planned. In contrast, novices appear trapped by cognitive tunneling [61], remaining stuck in a continuous, blended process of local editing without achieving a global, strategic perspective.

Finally, the growth curve analysis (Fig 3) provides a holistic view of this joint influence. Experienced translators’ production curves show a consistent and intense rhythm that adapts efficiently to constraints. The novices’ trajectory peaks in the Standard condition and fades in the Free condition, illustrating the Yerkes-Dodson Law [62] in action, which holds that performance follows an inverted-U relationship with arousal: both insufficient and excessive pressure impair functioning, with optimal performance occurring at a moderate level of activation. Again, the moderate deadline acted as an external scaffold, focusing novices’ cognitive resources; when this scaffold was removed, their less-developed internal strategies were exposed, leading to suboptimal performance. Thus, at the macro level, expertise manifests in the ability to strategically manage one’s cognitive resources, while novice performance is jointly determined by their underdeveloped self-regulatory control and the level of external pressure.

### 5.3. Rhythmic signatures as reflections of cognitive strategy

The distinct rhythmic patterns observed in this study (e.g., surface-level oscillations between fluent typing and longer pauses) may reflect behavioral signatures of the underlying cognitive strategies employed by translators. Interpreted through the lens of Knowledge Telling/Transforming as strategic orientations rather than discrete cognitive states, the results point to tendencies whereby expertise involves flexible coordination between more automated and more deliberative processing during drafting, alongside a strategic separation between Knowledge Telling- and Knowledge Transforming-oriented activities to regulate the overall translation process.

During drafting, experienced translators are broadly consistent with a more Knowledge Telling-oriented rhythm, which is evidenced by their stable segment length across all conditions (Table 3) and a resilient, intense production curve even under severe time pressure (Fig 3). This stability is a hallmark of automaticity [51,60,63], where the core process of retrieving linguistic equivalents and producing text has become so routinized that it consumes minimal cognitive resources. Such a cognitive efficiency thus frees them to handle severe time pressure without collapsing the production process. When time permits, their behavior increasingly aligns with a more deliberate Knowledge Transforming approach concentrated in the end revision phase, as shown by increased deletions and revision time. This involves a holistic re-evaluation and restructuring of the text’s meaning, style, and coherence.

The functional independence of drafting and revision phases is further supported by the absence of correlation between online and end-revision deletions (Table 7), indicating that these activities are not simply redistributed across phases but strategically organized. Their pausing behavior provides further insights into this coordination: long pauses, which may reflect planning and problem-solving, were weakly but significantly associated with lower end-revision effort (*r* = −.25), whereas mid pauses were moderately associated with more online revision (*r* = .54). These associations suggest that for experienced translators, LPs during drafting may have been effectively utilized to alleviate end-revision demands and MPs during drafting marked active local monitoring.

The cognitive benefit of functionally separating and effectively coordinating the drafting and end revision phases is underscored by the inherent language-switching cost in translation [39]. By developing a more automated drafting process, experienced translators minimize the cognitive load associated with switching between languages, thereby conserving cognitive resources to strategically invest in a focused, high-level Knowledge Transforming phase during revision, maximizing overall performance. Together, these patterns reflect an approach potentially characteristic of translation expertise: fluent, largely automated drafting punctuated by deliberate, problem-solving LPs, followed by a distinct revision phase. This phased approach evidences metacognitive control and efficient coordination of automatic and effortful processing.

In contrast, novices’ behavior suggests a continuous, blended Knowledge Transforming strategy even during drafting, evidenced by their relatively higher rate of online edits, unstable segment lengths, and moderately correlated online and end-revision deletion patterns (*r* = .45), indicating simultaneous generation and correction without a clear separation between the drafting and end-revision phases. From a cognitive-load perspective [64], simultaneously producing content, monitoring for errors, and considering global revisions may easily overload working memory, causing cognitive friction, frequent interruptions, and inefficient use of time.

This inefficiency also appears in novices’ pausing behavior. In effect, it was MPs rather than LPs during drafting that were weakly associated with lower final editing needs (*r* = −.23), suggesting MPs’ role as constant, cognitively intensive intervals that deviate from mere local monitoring, given that MP duration was not associated with online revision (*r* = 0) for novices. By contrast, LPs appeared comparatively underutilized (*r* = −.07) in reducing end-revision demands. Overall, novices seem to use MPs in a way that resembles the function of LPs among experienced translators—namely, to help reduce later revision effort—whereas LPs among novices may be underutilized or may simply reflect unstructured activity (e.g., mind wandering) rather than meaningful task-related processing, at least in the present study. This pattern aligns with existing studies’ [34–35] characterization of MPs as markers of high cognitive load in student translators, while also offering a more nuanced understanding of their role in local monitoring.

The effect of time pressure further exposes this lack of strategic control. Under high pressure (Short condition), novices are forced into a brittle Knowledge Telling mode; however, because the process is not automated, it is slow and inefficient. The moderate deadline appears to force novices into a more disciplined and expert-like rhythm (shorter segments, higher problem-solving LP density), helping them simulate the cognitive control they cannot yet manage internally.

In conclusion, novices have not yet developed the automaticity required for an efficient Knowledge Telling rhythm, nor have they acquired the strategic foresight to separate it from a dedicated Knowledge Transforming phase. Their rhythmic signature is one of a reactive process, making their performance highly dependent on the guiding hand of external constraints or scaffolding. Altogether, these findings portray novices’ process as a blended one: a reactive, high-load state where Knowledge Telling and Transforming are inefficiently mixed, resulting in a jagged, overlapping cycle of drafting and low-level editing rather than a deliberate, phased strategy.

Based on these findings, a key implication for professional practice, extending beyond translation to any complex writing or creative task, is the value of consciously separate automated (Knowledge Telling) from deliberated (Knowledge Transforming) modes of production. Practitioners can enhance efficiency by adopting a phased strategy, which minimizes the cognitive friction that arises from trying to create and critique simultaneously. The finding that a moderate deadline can act as an external scaffold for novices has clear educational value, suggesting that time conditions can be manipulated in a controlled classroom setting as a powerful pedagogical tool. Time-constrained (“speed training”) tasks can foster efficiency and acclimatize trainees to professional time pressure [65–66], while complementary untimed tasks can foreground deliberate, global revision strategies without the cognitive friction of simultaneous drafting. The ultimate goal is to help novices internalize the cognitive control and strategic flexibility that experienced practitioners display naturally, equipping them with the ability to adapt their cognitive rhythm to the demands of any given task.

## 6. Conclusions

This study systematically investigated how time constraints and translator experience interact to shape cognitive rhythm in the translation process, revealing distinct adaptive patterns at both micro and macro levels. The findings show that while some adaptations to time pressure are universal, experience is the critical factor that determines the strategic efficiency of these adaptations. Through multi-level keystroke logging analysis, we found that while all translators benefited from relaxed time constraints, showing increased production fluency and stability, only experienced translators exhibited evident strategic flexibility. They maintained consistent production rhythms across all time conditions and were able to strategically reallocate cognitive resources, distinctly separating the phases of drafting and revision. Novices, by contrast, showed a reactive, blended approach in which drafting and revision were inefficiently intertwined, and their process efficiency depended heavily on external scaffolding. These findings reveal that translation expertise is characterized not only by faster or more fluent output, but by metacognitive control: the ability to flexibly shift between automated, fluent text production and deliberate, effortful problem-solving and revision as task demands change. Novices, lacking this separation, are prone to cognitive overload and inefficient working patterns, particularly when time is unconstrained. These findings underscore the significance of strategic process management and developing conscious control over one’s cognitive rhythm in any text production or creative work.

While this study provides valuable insights, its conclusions should be considered in light of its limitations, which in turn pave the way for future research. The findings are based on a single, semi-specialized text type and one language pair. The cognitive rhythms observed might differ significantly with more creative literary texts, which may demand more Knowledge Transforming throughout, or with highly technical legal or medical documents that impose different cognitive loads. Future studies should extend this approach to other languages and text types to further elucidate how cognitive rhythm in translation adapts across contexts and expertise levels.

## Supporting information

S1 File Scores of the objective (a) and subjective (b) measures of text difficulty. (TIFF)
